# Medium-term real-world data for erenumab in 177 treatment resistant or difficult to treat chronic migraine patients: persistence and patient reported outcome measures after 17–30 months

**DOI:** 10.1186/s10194-022-01536-3

**Published:** 2023-01-16

**Authors:** Emma Troy, Arif A. Shrukalla, Alina Buture, Katie Conaty, Esther Macken, Roisin Lonergan, Jane Melling, Niamh Long, Eamonn Shaikh, Kieran Birrane, Esther M. Tomkins, Peter J. Goadsby, Martin H. Ruttledge

**Affiliations:** 1 Department of Neurology, Beaumont Hospital, Beaumont Road, Dublin 9, Ireland; 2 Dublin Neurological Institute, Department of Neurology, Mater Hospital, Eccles Street, Dublin 7, Ireland; 3 Independent Statistical Consultant, Wilton, Cork, Ireland; 4 NIHR SLaM Clinical Research Facility at King’s, King’s College London, UK and Department of Neurology, University of California, Los Angeles, CA Los Angeles, USA

**Keywords:** Resistant chronic migraine, Erenumab, Patient related outcome measures (PROM), Headache impact Test-6 (HIT-6), Migraine associated disability assessment (MIDAS) test, Migraine-specific quality-of-life questionnaire (MSQ)

## Abstract

**Background:**

Many migraine patients do not respond adequately to conventional preventive treatments and are therefore described as treatment/medically resistant or difficult to treat cases. Calcitonin gene-related peptide monoclonal antibodies are a relatively novel molecular treatment for episodic and chronic migraine that have been shown to be effective in short duration clinical trials in approximately 40–50% of all chronic migraine patients. Patient Related Outcome Measures (PROM) or Quality of Life (QoL) questionnaires are used to help measure response to treatment in migraine. Although some open label extension studies have become available for erenumab, there is a lack of real-world data pertaining to quality of life in the medium to long-term for chronic and treatment resistant migraine patients.

**Methods:**

A total of 177 treatment resistant CM patients were started on erenumab (70 mg or 140 mg subcutaneous injection every 4 weeks) in our three specialist Headache Clinics. Of these, 174 had their first injection between December 2018 and October 2019. All patients were evaluated with the following PROM: the Headache Impact Test− 6, Migraine Associated Disability Assessment test and Migraine-Specific QoL Questionnaire, before starting treatment with erenumab and at intervals of 3–12 months after starting treatment. The decision to continue treatment was based on subjective clinical improvement of at least 30% (as reported by the patient), supported with diaries and QoL questionnaires. We present here the QoL measurements for this group of 177 patients. Prior preventive migraine treatments included conventional oral prophylactic medications (such as topiramate, candesartan, propranolol, or amitriptyline), at least two cycles of PREEMPT protocol onabotulinumtoxin A or (in a small number of cases) neuromodulation with single pulse Transcranial Magnetic Stimulation.

**Results:**

Of the 177 patients who started treatment with erenumab, 68/177 (38.4%) stopped during the first year, either due to lack of efficacy (no significant benefit or only minimal improvement) and/or possible side effects. 109/177 (61.6%) patients reported clinically significant improvement after 6–12 months and wished to stay on treatment. Twelve of these 109 patients subsequently stopped treatment in the period between 1 year and up to June 2021 (mainly due to a worsening of their migraine). Therefore, a total of 97/177 patients (54.8%) remained on treatment as of June 2021 (duration of treatment 17–30 months, median of 25 months).

**Conclusion:**

Approximately 55% of treatment resistant or difficult to treat CM patients who trialled erenumab in our clinics reported a subjective benefit and were still on treatment after 17–30 months.

## Introduction

Headache disorders, predominately migraine, account for approximately 25–30% of all outpatient neurology referrals in Ireland and internationally [1]. Migraine represents a significant medical burden, both on an individual level and to society. The Global Burden of Disease study has ranked it as the second highest cause of years lived with disability worldwide, and the first cause for disability-adjusted life years for women aged 15–49 years [2]. The impact of migraine to the Irish Exchequer every year (population of more than five million people) is estimated conservatively to be €290 million [3]. The negative medical and societal effects of migraine extend beyond headache and are more pronounced in patients who have had at least one previous preventive treatment failure. For example, the My Migraine Voice global survey found that 85% of respondents reported negative aspects of living with migraine [4]. The term treatment resistant migraine is used to describe patients with persistent headache and associated migraine symptoms who fail to respond to multiple conventional migraine preventive treatments (typically three agents). However, there remains a lack of consensus on a precise definition [5].

Chronic migraine (CM) as classified by the ICHD-3 beta [6], represents a small subset of migraine patients who are impacted very negatively in terms of quality of life (QoL) and are frequently disabled. Medication overuse represents a significant risk for development of CM. This may cause resistance to conventional oral preventive treatments which can further complicate therapy in this group [7]. It is imperative that patients with CM are provided with effective treatment options and that such therapies have tolerable side effect profiles. More effective treatments would not only alleviate the suffering of individual patients, but it would also decrease the societal burden associated with CM. Over the last few years, it is has been proposed that erenumab (and the other three CGRP monoclonal antibody antagonists) should be considered as an early option for patients with treatment resistant or difficult-to-treat migraine, who have high unmet needs and few effective conventional treatment options [8].

Erenumab is a fully human anti-calcitonin gene-related peptide (anti-CGRP) monoclonal antibody [9]. It is an antagonist of the G protein-coupled-receptor (CGRPR) [10] and is licenced for the treatment of episodic and chronic migraine. It has demonstrated efficacy in short-duration clinical trials for reducing migraine frequency [9] and improving QoL outcomes [11].

We have evaluated real-world persistence and PROM/QoL outcomes in a cohort of patients with treatment resistant chronic migraine receiving erenumab in Ireland during a follow up period of 17–30 months. Given the results of previous clinical trials and more recent real-world studies, we hypothesised that erenumab would lead to improvement in QoL outcomes for treatment resistant chronic migraine patients. This type of medium-term data will hopefully complement and reinforce short-term clinical trial evidence in CM patients who do not respond adequately to conventional migraine prophylaxis.

## Methods

### Aim

We aimed to evaluate real-world PROM/QoL outcomes in a cohort of patients initiated on erenumab for treatment of resistant CM over a period 17–30 months.

### Setting

This multicentre retrospective audit was conducted across three regional specialist headache clinics in Dublin, Ireland.

### Design

This report summarizes persistence with treatment and PROM/QoL outcomes of patients treated with erenumab in a real-world clinical setting during a 17–30 month period of follow-up. This is the primary analysis of this data and the a-priori assumption was that at least 50% of patients would show a subjective response to treatment. No statistical power calculation was conducted prior to the study. It is an open-label, retrospective, observational audit (survey) performed as part of our routine clinical practice. QoL questionnaires were filled in by our patients just before starting treatment (from December 2018 onwards) and then periodically (in practice on average every 3–12 months) to the end of June 2021. Erenumab was kindly made available free of charge to our CM patients by Novartis through a Managed Access Program (MAP) in Ireland from November 2018 until the first half of 2022. The sample size was based on the available patients during this time period. Erenumab is now available in Ireland through the national public healthcare system since November 2021.

### Inclusion criteria

One hundred and seventy seven patients (≥ 18 years old) with difficult to treat or treatment resistant chronic migraine (ICHD-3 beta criteria) were evaluated. Patients were selected to commence treatment in three tertiary hospital-based Headache/Migraine Clinics in Dublin, Ireland. Patients are only included in this report if they failed (did not respond adequately and/or had significant side effects) at least three prior conventional migraine preventive treatments. However, almost all of our patients had failed at least four/five different migraine preventives. The prior failed treatments included oral prophylactic medications (such as topiramate, candesartan, venlafaxine, propranolol, nortriptyline or amitriptyline), PREEMPT Botox (onabotulinum toxin A, at least two treatment cycles three months apart), or neuromodulation with single pulse Transcranial Magnetic Stimulation (TCMS). Patients maintained on additional preventive or prophylactic migraine medication(s) or other preventive therapy were included in this audit. Patients were advised to limit acute medications (painkillers, NSAID’s and triptans) as much as possible and medication overuse was discussed where appropriate.

### Exclusion criteria

Patients were excluded from the audit analysis if they were on another anti-CGRP treatment (such as fremanezumab), were pregnant or planning a pregnancy within 6–9 months, had episodic migraine, had a latex allergy, or had a recent cardiovascular event (in the last 6–12 months).

### Data collection

The patients received either 70 mg or 140 mg erenumab every 28 days by subcutaneous injection. As part of our routine clinical practice, patients completed three migraine specific PROM/QoL questionnaires just before starting treatment with erenumab and at 3–12 month intervals, continuing for up to 17–30 months. The migraine specific QOL questionnaires used were: the Headache Impact Test-6, (HIT-6) [12, 13], Migraine Associated Disability Assessment (MIDAS) [14] and Migraine-Specific Quality-of-Life Questionnaire (MSQ) [15, 16]. The COVID-19 pandemic did compromise data collection and patient responses, as some patients had their hospital appointments postponed or had virtual consultations. However, sufficient data was still collected and sample sizes were sufficient for statistical analysis based on the available data.

### Ethics

Ethics approval was not required for this retrospective audit, as completion of migraine QoL questionnaires by patients is a routine part of our clinical practice. No additional patient data or information was collected, and specific investigations were not performed. Patients were informed that anonymised data would be collected, and verbal informed consent was obtained from all patients. This audit was registered in the hospitals where patients were seen.

### Statistical analysis

The data was analysed using baseline (time-zero) patient completed PROM/QoL questionnaire outcomes and then compared to scores at various time periods after starting treatment (1–6 months, 7–12 months, 13–18 months, 19–24 months, and 25–30 months). There were a number of missing data points as a consequence of delayed follow up due to the Covid-19 pandemic or incomplete questionnaires. Data was reported as observed and no imputation was carried out for missing data. From analysing the raw scores and reviewing histograms, we concluded that the data is unlikely to be normally distributed, and therefore median and inter-quartile ranges (IQR) for HIT-6, MIDAS, and MSQ scores were used for displaying the results. Frequencies of patients under analysis at each time point were also calculated. Calculations were carried out using RStudio 1.4.1106 and R 4.0.5 for Windows. Confidence intervals were set at 95% when comparing medians, and a significance level of *p* < 0.05 was used for two-tailed statistical analysis. Wilcoxon Signed Rank tests were used to compare the median scores across times. Mann-Whitney U tests were carried out to verify that ignored patients (i.e. those who did not record a score at both times being tested) did not systematically impact the tested median scores.

## Results

Persistence with erenumab treatment and PROM/QoL outcomes from 177 treatment resistant CM patients were collected over a 17–30 month period of follow-up in our clinics. Twenty nine men (16.4%) and 148 women (83.6%) were included, with a mean age of 42 and 43 years, respectively (see baseline demographics in Table 1). The median scores for the questionnaires, number of patients receiving treatment, and numbers completing each QoL questionnaires are shown in Tables 2, 3 and 4. Scores for each QoL questionnaire were grouped into six-month time intervals for statistical analysis. These tables also list the number of patients used for statistical testing versus those still on treatment, which in turn shows the number of missing data points at each time period. All 177 patients received at least one dose of erenumab (Time 0). By the end of the first evaluation period (1–6 months), 32/177 patients (18.1%) had discontinued treatment. By the end of the second evaluation period (7–12 months), a total of 68/177 patients (38.4%) had discontinued treatment. This represents persistence with treatment of 61.6% at 1 year. There were 109 and 97 patients remaining, respectively, in the third and fourth evaluation periods (13–18 and 19–24 months). A total of 97 patients remained on erenumab until we stopped collating the data in June 2021, representing a range of treatment of 17–30 months. Three of the 97 patients only had erenumab as far as the 13–18 month treatment period, 40 patients reached the 19–24 month treatment period and 54 patients had reached the last evaluation period of 25–30 months. Three patients did not fully complete their MIDAS questionnaires, so only 94 patients had recorded questionnaire data for this measure.

**Table 1:** Baseline Patient Demographics

	Total patients with CM included (*n = 177)*
**Sex**	
Male	29
Female	148
**Mean Age (SD)**	
Male	42 (12.4)
(19-62 yrs)	
Female	43 (13.7)
(18-74 yrs)

**Table 2:** Median HIT-6 score at baseline and follow up in months. Median difference in HIT-6 compared to baseline and percentage change in HIT-6 compared to baseline. Proportion of patients contributing to the data

Time	Baseline Median (Total)	Baseline Median (Tested)	HIT6 Median After	Change (%)	No. of patients completing questionnaires (No. of patients on treatment)
Zero	68	68			177 (177)
1 to 6	68	68	62	-6 (9%)	145 (161)
7 to 12	68	68	59	−9 (13%)	97 (145)
13 to 18	68	68	56	−12 (18%)	49 (109)
19 to 24	68	68	58	−10 (15%)	62 (97)
25 to 30	68	70	56	−14 (20%)	39 (54)

**Table 3:** Median MIDAS score at baseline and follow up in months. Median difference in MIDAS compared to baseline and percentage change in MIDAS compared to baseline. Proportion of patients contributing to the data

Time	Baseline Median (Total)	Baseline Median (Tested)	MIDAS Median After	Change (%)	No. of patients completing questionnaires (No. of patients on treatment)
Zero	130.5	130.5			174 (174)
1 to 6	130.5	138	55.5	−82.5 (60%)	133 (158)
7 to 12	130.5	119.5	30	−89.5 (75%)	90 (142)
13 to 18	126.5	119	24	−95 (80%)	49 (106)
19 to 24	129	123	27.5	−95.5 (78%)	60 (94)
25 to 30	123	115.5	14.5	−101 (87%)	40 (52)

**Table 4:** Median MSQ score at baseline and follow up in months. Median difference in MSQ compared to baseline and percentage in MSQ compared to baseline. Proportion of patients contributing to the data

Time	Baseline Median (Total)	Baseline Median (Tested)	MSQ Median After	Change (%)	No. of patients completing questionnaires (No. of patients on treatment)
Zero	63	63			177 (177)
1 to 6	63	63	46.5	−16.5 (26%)	135 (161)
7 to 12	63	63	41	−22 (35%)	93 (145)
13 to 18	62	57.5	36	−21.5 (37%)	48 (109)
19 to 24	63	61	38	−23 (38%)	55 (97)
25 to 30	61	66	36	−30 (45%)	35 (54)

**Fig. 1:**
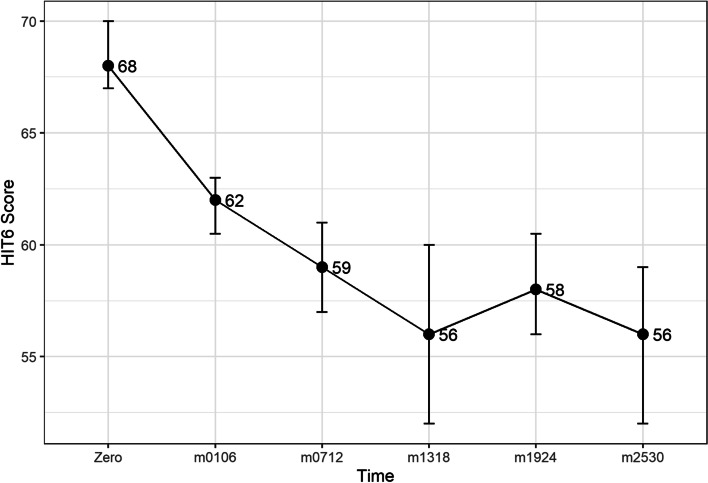
Median HIT6 score (y-axis) at baseline and up to 30 months of follow-up (x-axis). Ninety-five percent confidence interval of median shown by vertical bars

**Fig. 2:**
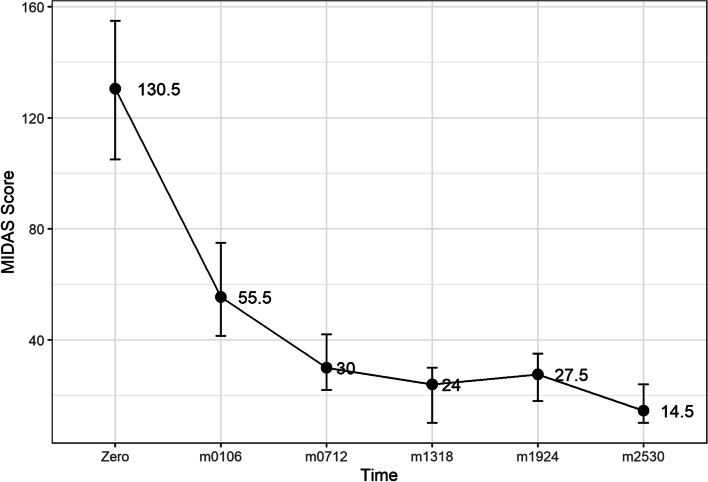
Median MIDAS score at baseline (y-axis) and up to 30 months of follow-up (x-axis). Ninety-five percent confidence interval of median shown by vertical bars

**Fig. 3:**
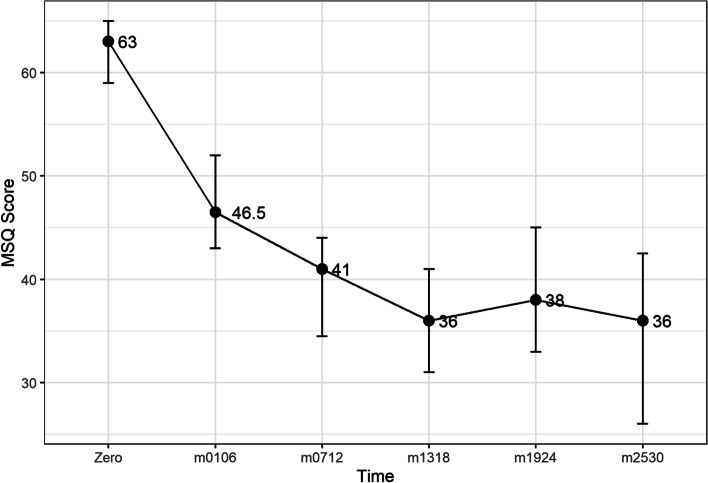
Median MSQ score at baseline (y-axis) and up to 30 months of follow-up. Ninety-five percent confidence interval of median shown by vertical bars

The change in median scores and the 95% confidence intervals are shown in Figs. 1, 2 and 3. Descriptive statistics of length of treatment and a summary of treatment length by patient group and time are shown in Tables 5 and 6 and Fig. 7. The change in median scores in absolute terms and percentage form are also recorded. All comparisons determined by the Wilcoxon Signed Rank test were statistically significant with *p*-values of < 0.001. All exclusions were tested using Mann-Whitney U tests, and were not statistically significant, apart from two tests: the MIDAS score comparison of baseline to time 1–6 months (*p* = 0.024), and the HIT-6 comparison of baseline to time 19–24 months (*p* = 0.047), though the latter showed no difference in median scores. The removal of patients who did not record time 1–6 month scores for MIDAS increased the baseline median from 130.5 to 138, suggesting that the reduction in score of 82.5 points (60%) may be slightly inflated.

**Table 5:** Descriptive statistics of the length of treatment per treatment group

Group	Min	1st Quartile	Median	Mean	3rd Quartile	Max
Continued	17	21	25	25.23	29	30
Stopped	1	6	8	8.61	11	24

**Table 6:** Number of patients per treatment group (Continued or Stopped by June 2021) per time period. Group Zero was determined to be patients who stopped treatment between months 1 and 5, as these were outside normal follow-up. Individual group totals and cumulative totals also included

Time (months)	Continued	Stopped	Total (individuals)	Total (cumulative)
Zero (< 6)	0	16	16	**16**
1 to 6 (=6)	0	16	16	**32**
7 to 12	0	36	36	**68**
13 to 18	3	9	12	**80**
19 to 24	40	3	43	**123**
25 to 30	54	0	54	**177**
**Total**	**97**	**80**	**177**	

**Fig. 4:**
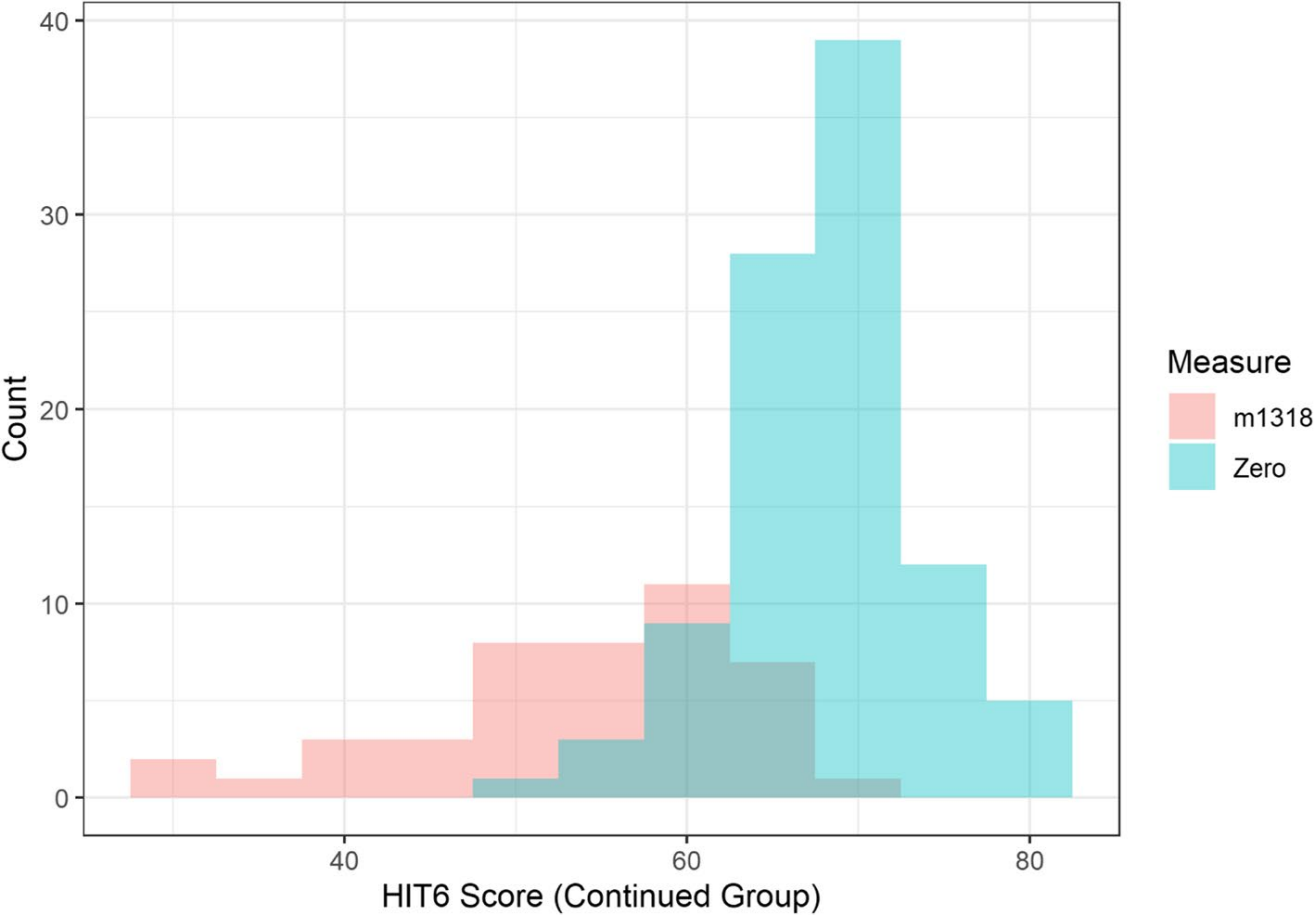
Histogram comparing times Zero and m1318 distribution of HIT6 scores for patients who remained on treatment beyond June 2021

**Fig. 5:**
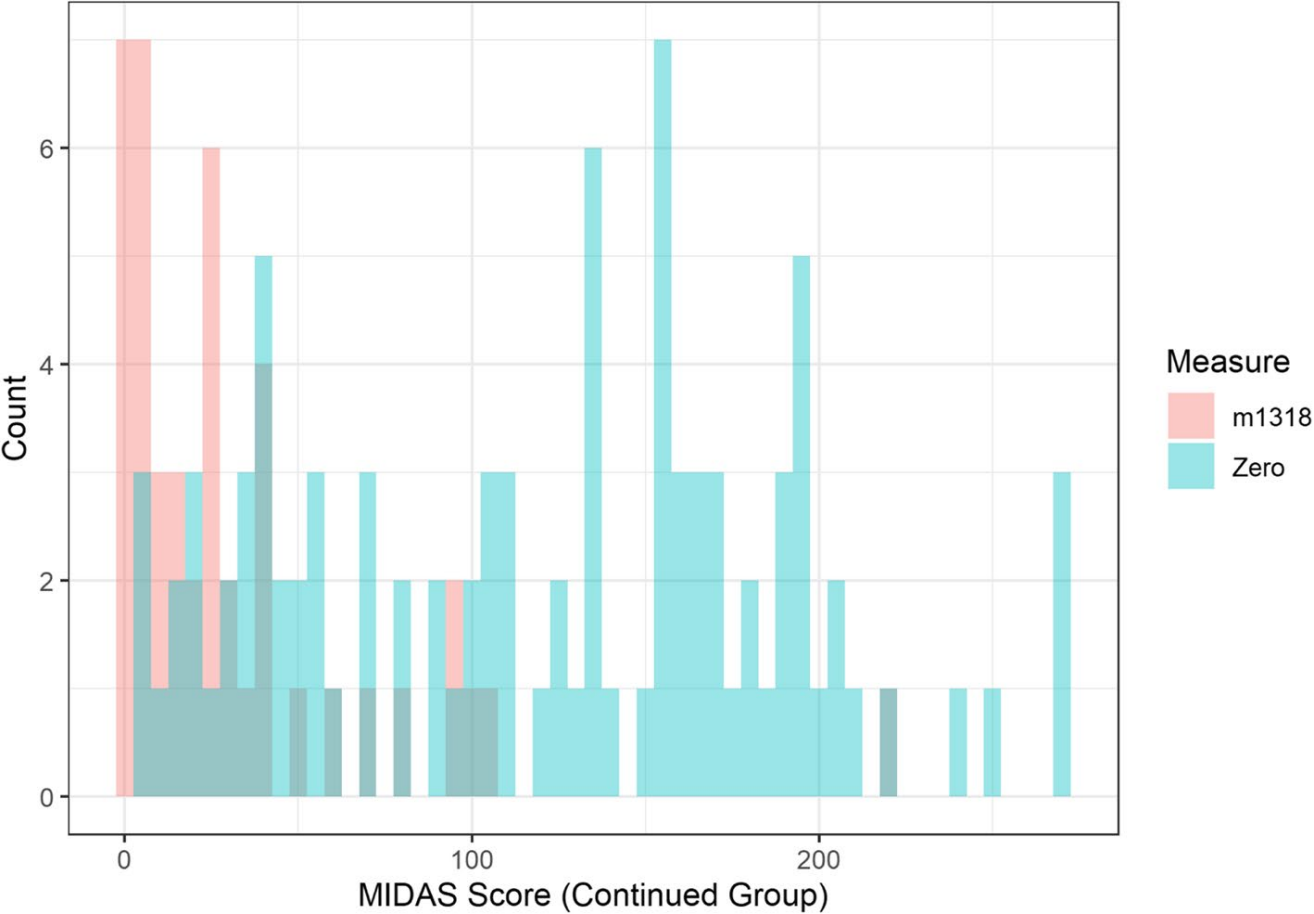
Histogram comparing times Zero and m1318 distribution of MIDAS scores for patients who remained on treatment beyond June 2021

**Fig. 6:**
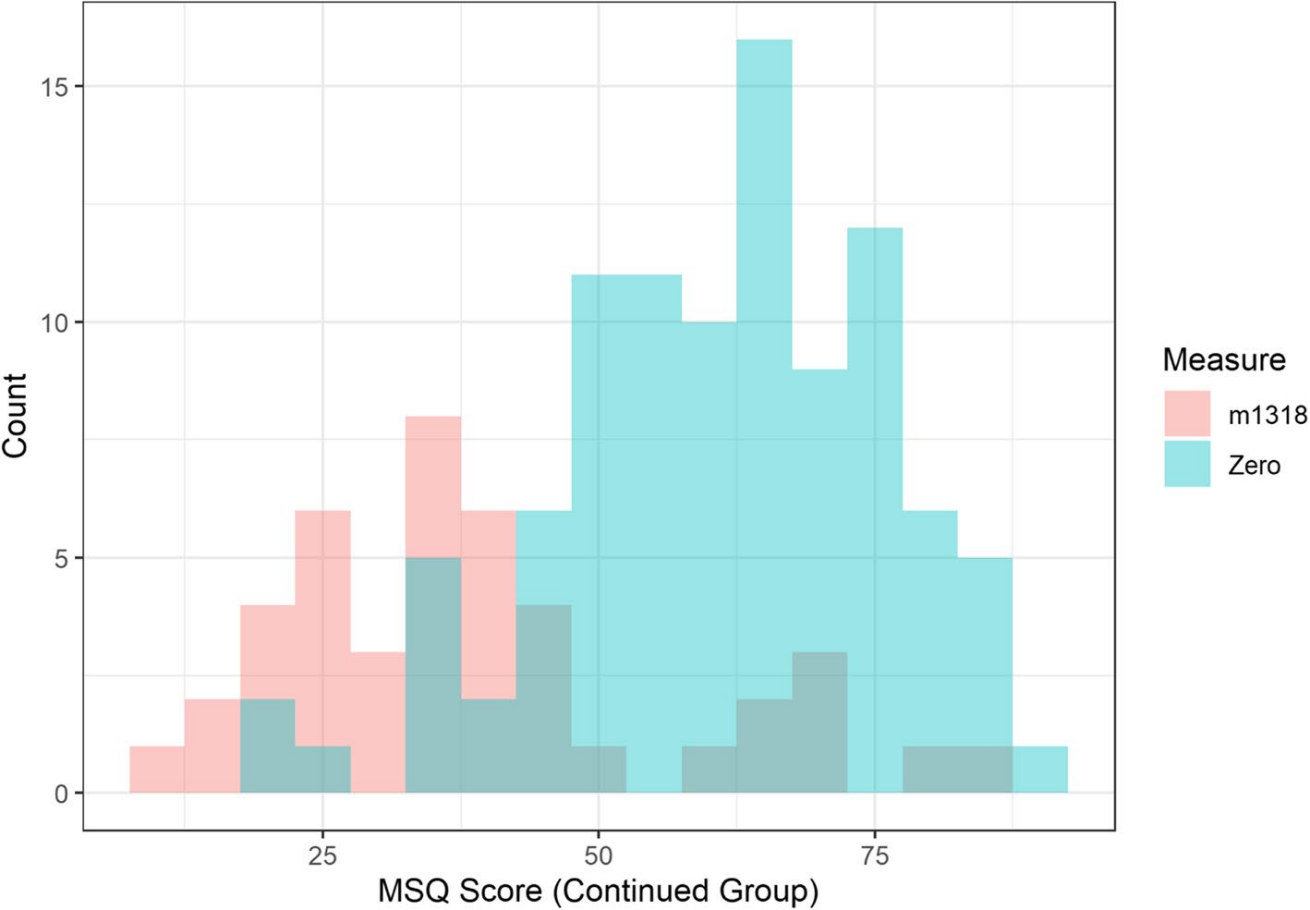
Histogram comparing times Zero and m1318 distribution of MSQ scores for patients who remained on treatment beyond June 2021

Patients who stopped treatment had been on treatment for between one and 24 months, with a median time of 8 months (mean = 8.6 months), and IQR of 5 (11-6). Patients who remained on erenumab until June 2021 had been treated for 17–30 months, with a median time of 25 months (mean = 25.2 months), and IQR of 8 (29-21). Histograms of the score for each of the QoL measures at time zero and at the time period 13–18 months for those who were still on treatment by June 2021 are shown in Figs. 4, 5 and 6. This time period was chosen for these figures as all patients who remained on treatment had data recorded within this time window and therefore it is a good visual depiction of the medium-term effects of erenumab in our patient cohort.

Of the 177 CM patients, 171 commenced on Erenumab 70 mg, whereas six patients commenced on 140 mg. This was a clinical decision based on body weight. Patients who weighed more than 100 kg were started on the double dose, whereas all other patients started on the lower dose. Of the patients who stayed on treatment beyond 3–6 months, all but four increased the dose to 140 mg, largely due to lack or response or inadequate response at the 70 mg dose. Four patients remained on the 70 mg dose for the duration of data recording due to excellent response at this lower dose.

**Fig. 7:**
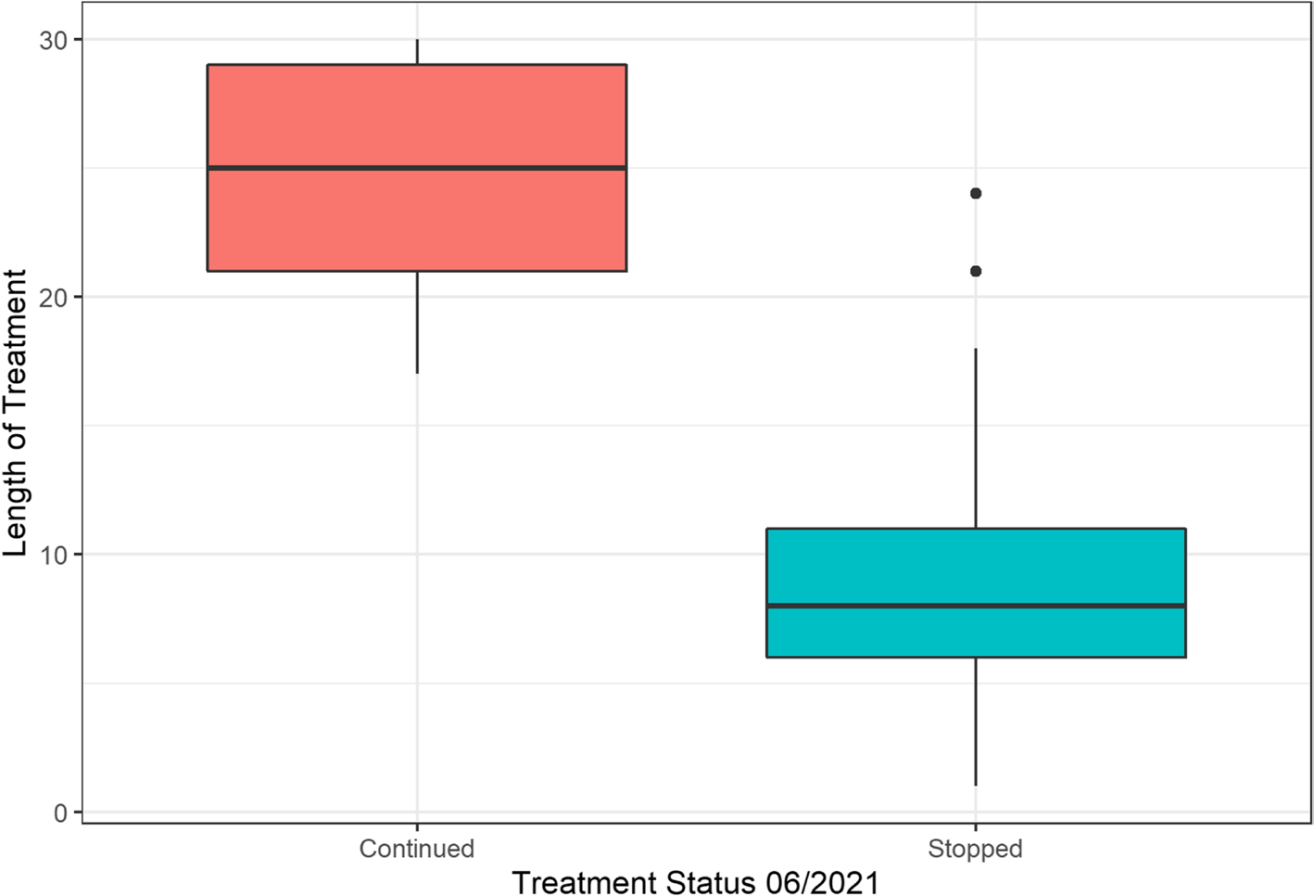
Boxplot comparing length of treatment between patients who stopped treatment vs patients who continued treatment

Discontinuation of erenumab treatment was mainly due to lack of efficacy (no significant or only minimal improvement) and less frequently, side effects. Some patients had modest improvement and mild/moderate side effects and therefore stopped because of both factors. The decision to continue treatment was based on subjective clinical improvement of at least 30% (as reported by the patient), in the absence of severe side effects, supported with diaries and QoL questionnaires. We divided patients into three groups. Group 1 (*n =* 97, continued for 17–30 months) had subjective benefit from erenumab (reporting at least 30% improvement) and had mild or no significant side effects. Group 2 (*n =* 5, discontinued) reported at least 30% subjective improvement but had moderate or severe side effects. Group 3 (*n =* 75, discontinued) deemed erenumab to be ineffective and had less than 30% improvement in their migraine headache and associated symptoms (with or without side effects). Figure 7 shows a boxplot comparing length of treatment between patients who stopped treatment versus patients who continued treatment.

Overall, PROM/QoL scores improved significantly over time when compared to baseline in those patients who remained on treatment. This effect was noted for all three measures used. The median reduction in HIT-6 compared to baseline ranged from 6 (by 6 months) to 14 points (after 24 months: 25–30 month group, 9–20%), indicating clinically significant improvement. The median reduction in MIDAS scores was comparable. Compared to baseline, the median reduction ranged from 82.5 to 101 points (60–87%), also indicating significant clinical improvement. Taking account of the 7.5 point median difference for time 1–6 months would reduce this to 75–101 points (54–87%). Median reduction in MSQ scores compared to baseline ranged from 16.5 to 30 points (26–45%). Patients did not generally report worsening of PROM/QoL measures while taking erenumab, although there were definite fluctuations in terms of magnitude of improvement over the full 17–30 month period of follow up. A significant proportion of patients had discontinued treatment after 12 months (almost 40%). Those who remained on treatment after 1 year generally reported further improvements in QoL scores over time (for example, median MIDAS was 30 at 7–12 months and 14.5 at 25–30 months). However, there were a further 12 patients who dropped out between the end of 1 year and June 2021 (after 17–30 months of treatment).

## Discussion

Emerging literature on patients with migraine treated with erenumab and other CGRP monoclonal antibodies outside of clinical trials has demonstrated improved outcomes for a significant proportion of cases [17–20]. We audited 17–30 months of PROM/QoL data in our cohort of treatment/medically resistant or difficult to treat CM patients to give us insight into the more medium to long-term benefits of anti-CGRP intervention in this type of patient population. During the first year, 68/177 (38.4%) patients stopped treatment mainly due to lack of efficacy (marginal or no improvement) and less frequently due to lack of efficacy and/or side effects. The reported side effects in our patients were generally mild, with the most common being injection site reactions, abdominal bloating and constipation. Two patients stopped erenumab after the first dose of 70 mg, both due to potentially more severe side effects. After 1 year, 109/177 (persistence of 61.6%) of patients continued treatment. By June 2021, 97/177 (persistence of 54.8%) had sustained clinical improvement and had remained on erenumab for 17–30 months (with 54 of these remaining on treatment for at least 25–30 months). This is a very significant proportion of patients showing improvement considering that all patients in this report had failed at least three conventional migraine preventive agents (typically these patients had cycled through 4–8 failed preventive treatments).

Persistence with preventive treatments in migraine in the medium to long term (1–3 years) is a good combined measure of efficacy and side effects. In a recent report from Canada, the persistence of erenumab in the medium term in more than 14,000 pooled EM and CM patients was approximately 71% at 1 year and 63% at 18 months [20]. This is slightly higher than in our patients (persistence of 61.6% at 1 year). This may be due in part to the treatment resistant nature of our patients. In addition, all of our patients had CM and the above study had a combination of EM and CM (with the assumption that EM patients are less treatment resistant). In contrast, it should be noted that the persistence of conventional oral preventive treatments for migraine (such as B-blockers, tricyclics, and anti-convulsant medications) is in the region of 20–30% after 1 year [21]. Given the data from numerous clinical trials, the more recent real-world studies referred to above and our data, it would appear that erenumab and the other CGRP monoclonal antibodies (fremanezumab, galcanezumab and eptinezumab) have superiority over conventional preventive treatments with better efficacy and less side-effects. However, it is early days as few direct head-to-head trials have been done. In this regard, the HERMES trial was the first randomised controlled trial comparing a conventional oral preventive agent (topiramate) to a CGRP antagonist (erenumab) in 777 migraine patients [22]. In support of the argument that CGRP antagonists are superior to conventional migraine preventive treatment, the investigators found that some 10% discontinued erenumab mainly due to adverse events compared to almost 40% in the topiramate group. In addition, significantly more patients in the erenumab group had a ≥ 50% reduction in monthly migraine days when compared with the topiramate group [22].

Erenumab has previously been shown to be beneficial for both treatment resistant EM and CM in short duration clinical trials. For example, the LIBERTY trial evaluated the efficacy of erenumab in patients with episodic migraine who failed 2–4 other prophylactic agents [23]. After 12 weeks, approximately 30% of those receiving erenumab 140 mg had achieved a ≥ 50% reduction in migraine days, compared to 13.7% for the placebo group [8, 9]. Long term outcomes from the LIBERTY study over a 64-week period highlighted persistent efficacy and safety of erenumab. In an open label extension study, sustained efficacy and safety with erenumab over a five-year period has been demonstrated [24]. Given our findings and those of others more recently, further studies looking at longer term outcomes are needed to evaluate more meaningful clinical response in a real-world setting. However, in this report, we provide real-world data for 17–30 months of treatment with erenumab which has not been previously documented in such a group of difficult to treat or treatment resistant CM patients.

In our evaluation, we included the HIT-6, MIDAS and MSQ questionnaires. These Patient Reported Outcome Measures (PROM) are frequently used and internationally accepted instruments for this cohort of patients. For example, a subgroup analysis of a double-blind trial of those with CM and medication overuse headache (MOH) comparing erenumab 70 mg or 140 mg to placebo, utilised MIDAS, HIT-6 and MSQ [11]. In this study, erenumab demonstrated improvement in PROM/QoL and disability using these tools. These findings are consistent with other recently published real-world evidence [18, 19].

A clinically meaningful or minimally important difference on the HIT-6 was considered to be ≥6-point improvement [25]. Clinically meaningful or minimally important difference for the MIDAS and MSQ questionnaires are not as clear cut and there is currently no expert consensus (although individual groups have suggested criteria). However, our data demonstrated a similar reduction in MIDAS and MSQ scores from baseline that mirrors the HIT-6 data. The decision to continue treatment was based on a subjective clinical improvement of at least 30% (as reported by the patient): this decision was reinforced by headache diaries and PROM/QoL questionnaires completed by our patients. Approximately one fifth of patients were deemed to be ‘super-responders’, as they had an overall clinical improvement in the region of 80–90% or more.

There are currently no biomarkers or diagnostic tests available to assess whether a particular migraine treatment is helpful for patients, and therefore QoL questionnaires, diaries and patient consultations are currently the only reliable ways to assess for meaningful clinical improvement. There are a number of known limitations when using QoL questionnaires or PROM in CM. One factor is that none of the QoL questionnaires used in our analysis have been specifically designed for patients with CM. They are “migraine specific questionnaires” and the majority of patients with migraine have EM. This needs to be taken into account as significant differences exist between groups of EM and CM patients. For example, patients with CM have a significantly higher level of disability than patients with EM [26]. Another stumbling block is that the MSQ questionnaire was designed to assess QoL attributable to migraine in patients who were in a non-migrainous state [15]. This is certainly not the case for our cohort of patients as, by definition, CM patients have headache and migraine symptoms on more days per month than not. In fact, many of our patients had daily symptoms and few or no crystal-clear days. In support of our methodology, there are several studies in the last ten years that have demonstrated a reasonable level of legitimacy of use of these QoL questionnaires in CM. For example, Rendas-Baum, Bloudek [27] demonstrated the validity of MSQ in assessing QoL outcomes in CM patients. This study confirmed further credibility by demonstrating strong associations with HIT-6. The recently published erenumab trials included the newly developed Migraine Physical Function Impact Diary (MPFID) scale, a PROM which is designed to assesses the impact of migraine physical function over the previous 24 hours and 7 days [26]. We did not use this questionnaire in our study as it is not currently widely accepted or routinely used in clinical practice. It may have been useful to compare our patient group to studies using this parameter [28]. The most important clinical factors for patients relating to treatment include reducing disability, improving quality of life, increasing workplace productivity, reducing presenteeism, and being able to function in their daily activities. In this regard, PROM/QoL questionnaires can be used as an additional resource to improve our understanding of the impact of certain therapies for our patients with greater disability and to monitor evolution on treatment over time [25].

It is generally accepted that CM cohorts as a subgroup represent a complex and heterogenous group of patients. There are various factors which predispose patients to evolve from EM to CM. Risk factors for chronicity include: age, female sex, obesity, depression, low educational status and medication overuse [7, 29]. There is also clinical evidence that genetic factors make some individuals prone to developing more chronic forms of migraine. From a treatment perspective, there is preliminary data that individual responses to anti-CGRP therapies in migraine may be dependent on epigenetics [30]. From our small cohort and emerging evidence in the literature, it is clear that there is significant individual variation in terms of response to CGRP blocking treatment. Interestingly, certain patients do not experience any improvement whatsoever, while others are ‘super-responders’ and achieve a near complete resolution of their migraine headache and associated neurological symptoms.

All CM patients in this study had failed at least three conventional migraine preventive treatments prior to starting erenumab and are therefore considered to be medically resistant, or difficult to treat migraineurs. This more resistant group is of particular interest for us as anti-CGRP therapies have recently been approved for reimbursement in the Irish healthcare system specifically for those who have failed at least three conventional options. This is consistent with several other healthcare systems internationally.

Many of our 177 erenumab patients opted to remain on various additional migraine preventive therapies for all or part of the 17–30 month treatment period (including onabotulinumtoxin A injections or oral prophylaxis), perhaps partly because they were having some perceived additional benefit with these non-CGRP therapies. We have therefore wondered if there may be some clinical synergy with respect to these different treatments. In a recent review in *Headache*, it has been proposed that there may be a pathophysiological rational to dual prescribing of onabotulinumtoxinA and anti-CGRP agents, as there is a possibility of clinical synergism [31]. However, this symbiotic relationship has not yet been confirmed at the time of writing, and there is currently only preclinical evidence to support a rational for dual therapy. It may be worthwhile in future studies to address whether patients being treated with additional preventive treatments had incremental improvement in QoL outcomes compared to those on CGRP treatment alone.

The disability associated with migraine should not be underestimated. When comparing migraine to other neurological disorders, it is the second cause of neurological impairment worldwide next to stroke [32]. In a disease burden study of over 11,000 participants for whom preventive treatments have failed, 52% reported impairment in both overall work productivity (absenteeism and presenteeism combined) and daily activities due to migraine [4]. In the same study, 64% of respondents reported that migraine had affected their private life, including relationships with friends, relatives, and partners. It is well documented that loss in work productivity and activity impairment due to migraine is higher in individuals with previously failed preventive treatments, further establishing the need for more effective treatment options in this subpopulation to ensure that they can fully contribute to the workforce and to society [4].

## Conclusion

Our retrospective real-world audit of erenumab in patients with medically treatment resistant CM, provides evidence of clinical improvement in approximately 55% of cases. Our data therefore demonstrates that erenumab treatment provides patient-reported improvement in QoL in a significant proportion of treatment resistant CM cases in the medium term. These findings appear to be robust and consistent across the three instruments that we used in our clinical practice (MIDAS, HIT-6 and MSQ). Understanding the benefit of CGRP treatments in different groups of migraine patients is important for clinical decision making, along with informing national policy for treatment optimisation for patients with migraine, particularly those who have failed at least three standard preventive therapies.

## Data Availability

The datasets used and/or analyzed during the current audit are available from the corresponding author on reasonable request.
